# Pancreatic Heterotopia of the Gallbladder: A Rare Phenomenon That Might Be on the Rise

**DOI:** 10.7759/cureus.81355

**Published:** 2025-03-28

**Authors:** Hugo Cohen, Harry Haynes, Darko Lazic, James Williamson

**Affiliations:** 1 Royal Centre for Defence Medicine, Academic Department of Military Surgery and Trauma, Birmingham, GBR; 2 Department of General Surgery, Great Western Hospital, Swindon, GBR; 3 Department of Cellular Pathology, Great Western Hospital, Swindon, GBR

**Keywords:** general surgery, histology, incidental histopathological findings, laparoscopic cholecystectomy, pancreatic heterotopia

## Abstract

A young patient presenting with recurrent biliary colic had a laparoscopic cholecystectomy. The routine histology on the gallbladder revealed a section of pancreatic tissue in the neck of the gallbladder. Pancreatic tissue separate from the pancreas is known as pancreatic heterotopia and is particularly rare. However, there has been a recent increase in the publications of cases. In this report we present the case history of our patient and discuss it with relevance to the wider literature.

## Introduction

The presence of pancreatic tissue within the gallbladder is a rare congenital finding, with 57 cases reported in the literature [1,2]. The incidence of disease seems to be increasing, but the reason for this is uncertain. Most of these cases are noted incidentally, as preoperative detection is impossible and may be reported as gallbladder polyps. Most patients undergo cholecystectomy for symptomatic improvement for concurrent stones, cholecystitis, or polyp removal. Histological examination of gallbladders may reveal a macroscopic focus of pancreatic heterotopia and will determine the location and extent of the disease. This case report will describe our case and discuss it with relevance to the published literature.

## Case presentation

**Figure 1:**
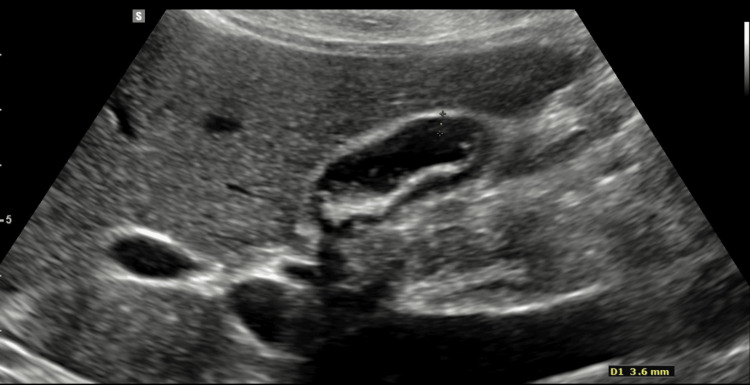
Patient's ultrasound image of thin-walled gallbladder containing stones

A 32-year-old woman presented with classical biliary symptoms of upper abdominal pain that radiated to the chest, accompanied by postprandial nausea. She had been complaining of intermittent sickness over the previous five years, with normal endoscopy, abdominal ultrasound (including for gallstones at that time), and computed tomography imaging. On admission, initial blood tests were unremarkable, and abdominal ultrasonography confirmed a thin-walled gallbladder containing stones (Figure 1) and an otherwise normal biliary tree. She was managed conservatively with oral antibiotics, analgesia, and antiemetics and discharged pending an elective laparoscopic cholecystectomy. This is standard care at our institution.

Three weeks later, she presented with worsening pain and diarrhea; clinical examination noted right upper quadrant tenderness, but no signs of sepsis. Blood tests were unremarkable. During this subsequent admission, she underwent a "hot" laparoscopic cholecystectomy three days later. At operation, an inflamed gallbladder was identified, but the gallbladder was removed via a standard four-port technique after achieving the critical view of safety. She made a good postoperative recovery and was discharged the following day.

**Figure 2:**
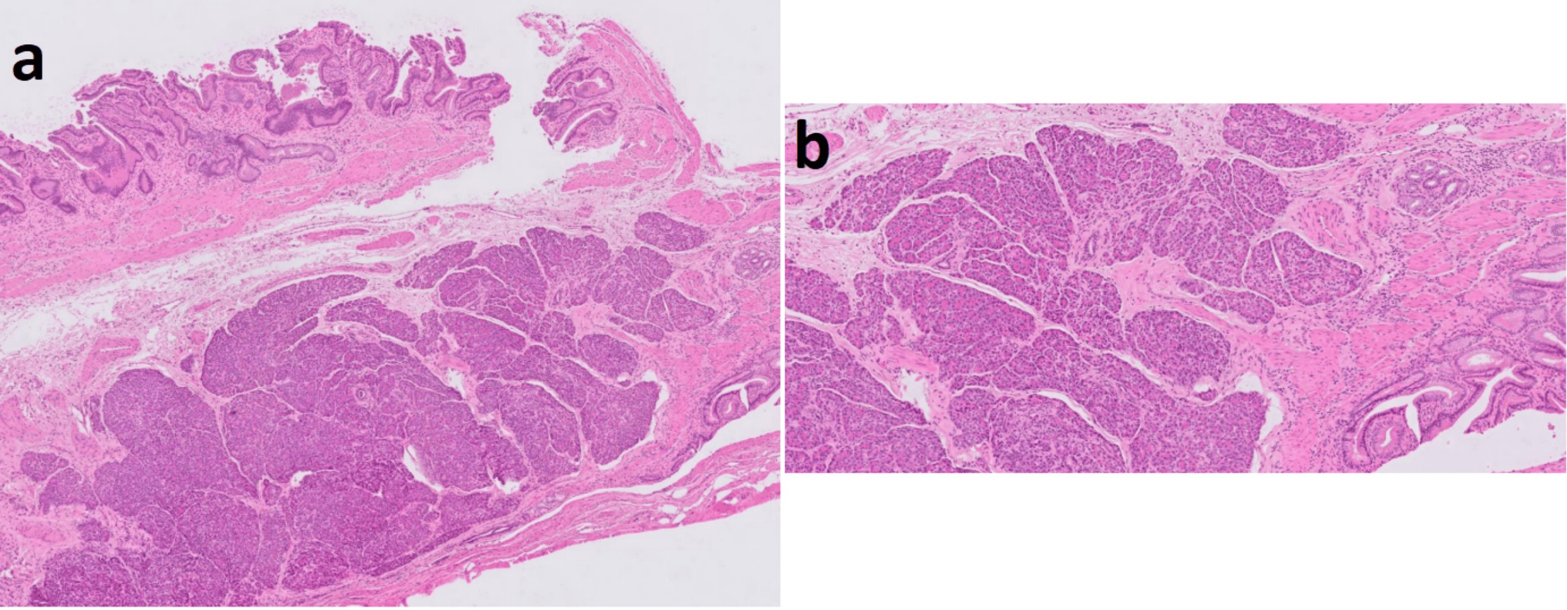
(a) A low-power view of the gallbladder neck shows mucosal cholesterolosis with mural muscular hypertrophy and some Rokitansky Aschoff sinus formation. These changes are of mild chronic cholecystitis. Underlying this are mural aggregates of pancreatic acini with occasional pancreatic ducts, seen at higher power in (b). No endocrine pancreatic components are identified. These features are in keeping with a focus on pancreatic heterotopia within the neck of the gallbladder (H&E sections) H&E: hematoxylin and eosin

Histological examination (Figure 2) of the specimen reported changes of mild chronic cholecystitis. There were subserosal aggregates of pancreatic acini and occasional pancreatic ducts within the neck of the gallbladder, but no evidence of endocrine islets. There was no evidence of atypia or malignancy.

## Discussion

Pancreatic heterotopia is a congenital abnormality defined as abnormally located pancreatic tissue not connected to the pancreas by vascular or other means. It is typically located in the stomach, small intestine, or in a Meckel’s diverticulum, and rarely in other sites, including the gallbladder. Two main theories exist for the presence of pancreatic heterotopia: firstly, that primitive pancreatic tissue is detached during embryological rotation of the visceral contents or that fragments are detached during longitudinal growth of intestines [1]. Secondly, it is due to an error in the Notch signaling system that decides the fate of pancreatic cells: this is due to an error in the Hes-1 (hairy and enhancer of split-1) cells in gallbladder pancreatic heterotopia [2].

The first report of heterotopic pancreatic tissue was by Schultz et al. in the eighteenth century [3], and histological classification followed by Heinrich et al. and later refined by Fuentes in 1973 to a classification of four types depending on the histological appearance of the pancreatic tissue [2]. The features identified in this patient’s specimen are in keeping with a focus of pancreatic heterotopia, with a modified Henrich classification type 1 (Table 1).

**Table 1:** Modified Heinrich classification for pancreatic heterotopia (+ve = positive, -ve = negative) Adapted from Serboiu et al., 2023 [1]; open access

Type	Histological features	Immunohistochemical features
1	Pancreatic acini, pancreatic ducts, endocrine islets	Cytokeratin 8/18, 19 +ve chromogranin/insulin +ve
2	Pancreatic ducts	Cytokeratin 8/18, 19 +ve chromogranin/insulin -ve
3	Pancreatic acini	Cytokeratin 8/18, 19 +ve chromogranin/insulin -ve
4	Endocrine islets	Cytokeratin 8/18, 19 -ve chromogranin/insulin +ve

Fifty-seven cases have been reported within the literature, and there seems to be an increasing rate of occurrence with 15 cases published over the last four years [1,4-17], since the largest literature review [2]. The reason for the increasing incidence is not clear, but it is thought to be more likely due to better recognition of the disease rather than a true increase in cases. As in our case, there is a female predominance, but this may reflect the higher number of cholecystectomies performed in women [2]. The mean age of diagnosis is 43.6 (range 8-80), and in most cases, cholecystopathic symptoms were the cause for operation, with cholelithiasis present in approximately 50% of cases. Preoperative imaging does not routinely identify ectopic pancreatic tissue; when abnormalities are noted, they tend to be reported as polyps or malignancy [2].

Pancreatic heterotopia can be located in any region of the gallbladder, with a subtle predominance for the neck, with approximately 40% of cases noted within this region. Histological examination of the ectopic tissue can note exophytic polypoid growth or yellow-colored nodules that can range from a few millimeters to four centimeters in maximal diameter [2]. In our case, the mucosal surface of the gallbladder was unremarkable, and the ectopic tissue was only noted on a routine section of the specimen. One case within the literature has noted pancreatic heterotopia alongside adenocarcinoma [15].

The relative paucity of evidence within the literature would suggest that gallbladder pancreatic heterotopia is an incidental finding with little consequence on patient management. However, the presence of ectopic tissue within the gallbladder neck in particular may predispose patients to acalculous cholecystitis or carcinoma [1]; conceivably, the ectopic tissue may also limit the flow of bile from the gallbladder, concentrating the remaining fluid and predisposing to calculi precipitation. It is not known if episodes of pancreatitis cause subsequent inflammation of the ectopic tissue within the gallbladder. The presence of amylase from this gallbladder pancreatic heterotopia at the cystic duct, in particular, may be a cause of concern for surgeons, as this virulent enzyme may predispose the patient to a prolonged recovery or, at worst, a bile leak.

Our patient had been extensively investigated over the preceding five years for symptoms of vomiting. Her gallstones had been noted on imaging during a flare-up of pain. Although it is possible that the pancreatic ectopic tissue was an incidental finding, the history and previous negative scans allude to the fact that pancreatic heterotopia may be related to her symptoms. Conceivably, inflammation of the ectopic tissue may be causing gallbladder irritation and inflammation of the surrounding tissue (which could be affecting her gastric outlet), predisposing to nausea and vomiting. Alternatively, the presence of ectopic tissue in the neck of the gallbladder may affect bile flow with resultant precipitation of calculi. In retrospect, it is difficult to predict if knowledge of the pancreatic heterotopia would have affected management, either in the form of endoscopic ultrasound to try and visualize the gallbladder neck in more detail, or if cholecystectomy would have been indicated without either stones or classical biliary symptoms.

## Conclusions

We report an incidental finding of gallbladder pancreatic heterotopia within the neck of the gland. This case is unusual as macroscopic examination of the gallbladder was unremarkable, and the abnormality was only noted on routine microscopic assessment. As the incidence of this ectopic disease within the gallbladder is rising, both in part due to increased numbers of cholecystectomies being undertaken and better recognition of the disease, it is conceivable that the true prevalence of the condition is underreported. If the ectopic tissue can only be noted on routine microscopic examination, as in this case, then it may be frequently missed or overlooked. Whether an increased awareness of pancreatic heterotopia within the gallbladder will be beneficial is uncertain, given that preoperative diagnosis is unlikely, and it does not seem to affect management (either in the rationale for cholecystectomy or intraoperative decision-making).
